# Optical-Helicity-Dependent Orbital and Spin Dynamics in Two-Dimensional Ferromagnets

**DOI:** 10.1021/acs.jpclett.4c01152

**Published:** 2024-05-29

**Authors:** Shuo Li, Ran Wang, Thomas Frauenheim, Junjie He

**Affiliations:** † Institute for Advanced Study, Chengdu University, Chengdu 610106, China; ‡ School of Science, Constructor University, Bremen 28759, Germany; § Department of Physical and Macromolecular Chemistry, Faculty of Science, Charles University, Prague 12843, Czech Republic

## Abstract

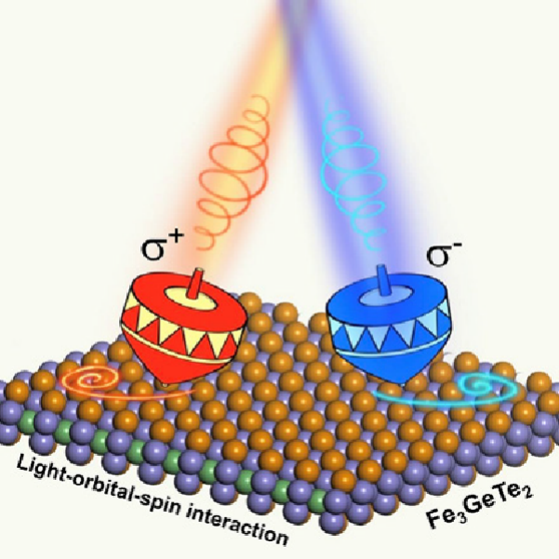

Disentangling orbital (OAM) and spin (SAM) angular momenta in the ultrafast spin dynamics of two-dimensional (2D) ferromagnets on subfemtoseconds is a challenge in the field of ultrafast magnetism. Herein, we employed a non-collinear spin version of real-time time-dependent density functional theory to investigate the orbital and spin dynamics of the 2D ferromagnets Fe_3_GeTe_2_ (FGT) induced by circularly polarized light. Our results show that the demagnetization of the Fe sublattice in FGT is accompanied by helicity-dependent precession of the OAM and SAM excited by circularly polarized lasers. We further identify that precession of the OAM and SAM in FGT is faster than demagnetization within a few femtoseconds. Remarkably, circularly polarized lasers can significantly induce a periodic transverse linear response of the OAM and SAM on very ultrafast time scales of ∼600 attoseconds. Our finding suggests a powerful new route for attosecond regimes of the angular momentum manipulation to coherently control helicity-dependent orbital and spin dynamics in 2D ferromagnets.

Ultrafast manipulation of the spin degree of freedom in magnetic materials serves as a fundamental principle for both storing and reading magnetic information using laser pulses.^1−4^ In addition to advancing our knowledge of fundamental physics, this research holds the promise of developing faster, more efficient, and more reliable magnetic storage devices and exploring new ways to exploit spin-related phenomena in future technological applications,^5−8^ with a focus on understanding the underlying physical processes of ultrafast demagnetization and spin switching in magnets.^9−11^ In two-dimensional (2D) magnetic materials,^12,13^ a remarkably efficient exchange between the orbital and spin angular momentum can take place due to the influence of spin–orbit coupling (SOC) resulting from the relativistic motion of the electrons. Distinguishing the individual contributions of the orbital and spin angular momentum in the 2D limit is a considerable challenge, mainly due to their rapid interplay, which occurs over a few femtoseconds.^14−17^

The advent of femtosecond and attosecond time scales, made possible by ultrashort optical laser pulses, offers new insights into ultrafast physics.^14,18^ By carefully studying the ultrafast dynamics induced by laser pulses, we can gain a deeper understanding of the transfer mechanisms and dynamics between orbital and spin degrees of freedom in 2D magnetic materials.^13,19^ All-optical-helicity-dependent switching (AO-HDS)^20,21^ with the right circularly polarized and left circularly polarized pulses can switch magnetization without any external magnetic fields. The ultrafast circularly polarized lasers can switch the magnetization in 2D magnetic systems, such as WSe_2_–CrI_3_ van der Waals heterostructures.^22^ However, the underlying mechanism of AO-HDS is still not unclear. AO-HDS could be generally explained by the inverse Faraday effect^23−25^ and the magnetic circular dichroism.^26−28^ Although previous investigations have provided some potential physical images for the dynamics of the angular momentum and demagnetization in the AO-HDS process,^29−31^ the interactions between light, orbital, and spin are much more complicated and difficult to understand at a few femtoseconds. The microscopic mechanism of direct light–orbital–spin interactions so far is still not clear and needs to be further explored in a few femtoseconds.

Real-time time-dependent density functional theory (rt-TDDFT) can successfully describe the linearly polarized laser-induced demagnetization.^32^ Dewhurst et al.^33^ showed the early spin dynamics based on the optical intersite spin transfer (OISTR) effect. This method allows us to study the spin dynamics and even the angular momentum in a few femtoseconds and even to distinguish the details on the attosecond time scales. Moreover, circularly polarized lasers have triggered the generation of an orbital angular momentum in the nanoparticle^34^ and explored the helicity-dependent spin dynamics of Fe, Co, and Ni from the optical regime to the extreme ultraviolet regime.^35^ Recently, the attosecond magnetization dynamics in the 2D nonmagnetic BiH driven by intense femtosecond lasers has been theoretically proposed,^36^ where the optical response is enabled by transverse light-driven currents and typically occurs on time scales of ∼500 attoseconds. Consequently, the study of orbital and spin dynamics and the exploration of the underlying mechanism of directly coherent light–orbital–spin interactions in 2D magnets will open new avenues for the development of ultrafast spintronics driven by circularly polarized light.

The 2D ferromagnets (FMs) Fe_3_GeTe_2_ (FGT)^12,37^ allow us to explore light–matter interactions in the 2D limit, due to their interesting physical phenomena.^38,39^ FGT could display tunnelling magnetic Skyrmions,^40,41^ high spin–orbit torques,^42^ and strong electron correlation effects.^43,44^ Furthermore, the magnetic properties of FGT can be tuned with laser light. For example, Liu et al.^19^ reported that the magnetic anisotropy of a few-layer FGT can be manipulated by using a femtosecond laser pulse. The previous work showed that laser pulses induce significant large spin injection from FM FGT into nonmagnetic (NM) layers within a few femtoseconds, where an interfacial atom-mediated spin transfer pathway in NM–FM heterostructures was identified.^45^ Recently, Li et al.^46^ reported that the intralayer spin transfer in graphene–FGT and silicence–FGT heterostructures could be controlled by terahertz lasers. These novel physical properties of FGT suggest that research on the photoinduced dynamic process in FGT could lead to the development of a new generation of ultrafast spintronic devices. However, the nature of the angular momentum flow and transfer between light, orbital, and spin during ultrafast demagnetization phenomena remains elusive in the 2D regime. Therefore, the study of direct coherent interactions between light, orbit, and spin on a few femtoseconds in FGT would be interesting and challenging.

In this work, we have investigated the orbital and spin dynamics in FGT excited by circularly polarized laser pulses using the non-collinear spin version of rt-TDDFT. The results show that the circularly polarized lasers can lead to different orbital and spin dynamics in the demagnetization processes, due to the linear response of the light–orbital–spin and the OISTR effect. Furthermore, the angular momentum transfer between the angular momentum of the polarized fields and the orbital angular momentum occurs on a few hundred attosecond time scale. This helicity-dependent orbital and spin dynamics in 2D magnetic materials would open a new way to manipulate the angular momentum for ultrafast spintronics.

The atomic structures of the optimized FGT monolayer are shown in Figure 1a. The lattice parameters of the unit cell of FGT are *a* = *b* = 4.051 Å. Three Fe atoms in the unit cell are located in two inequivalent sites, shown as Fe_1_/Fe_2_ and Fe_3_. Each monolayer FGT consists of five sublayers (Figure 1b). The FGT is metallic, and the strong spin–orbital coupling (SOC) in Fe atoms leads to the out-of-plane magnetocrystalline anisotropy.^12,19^

**Figure 1:**
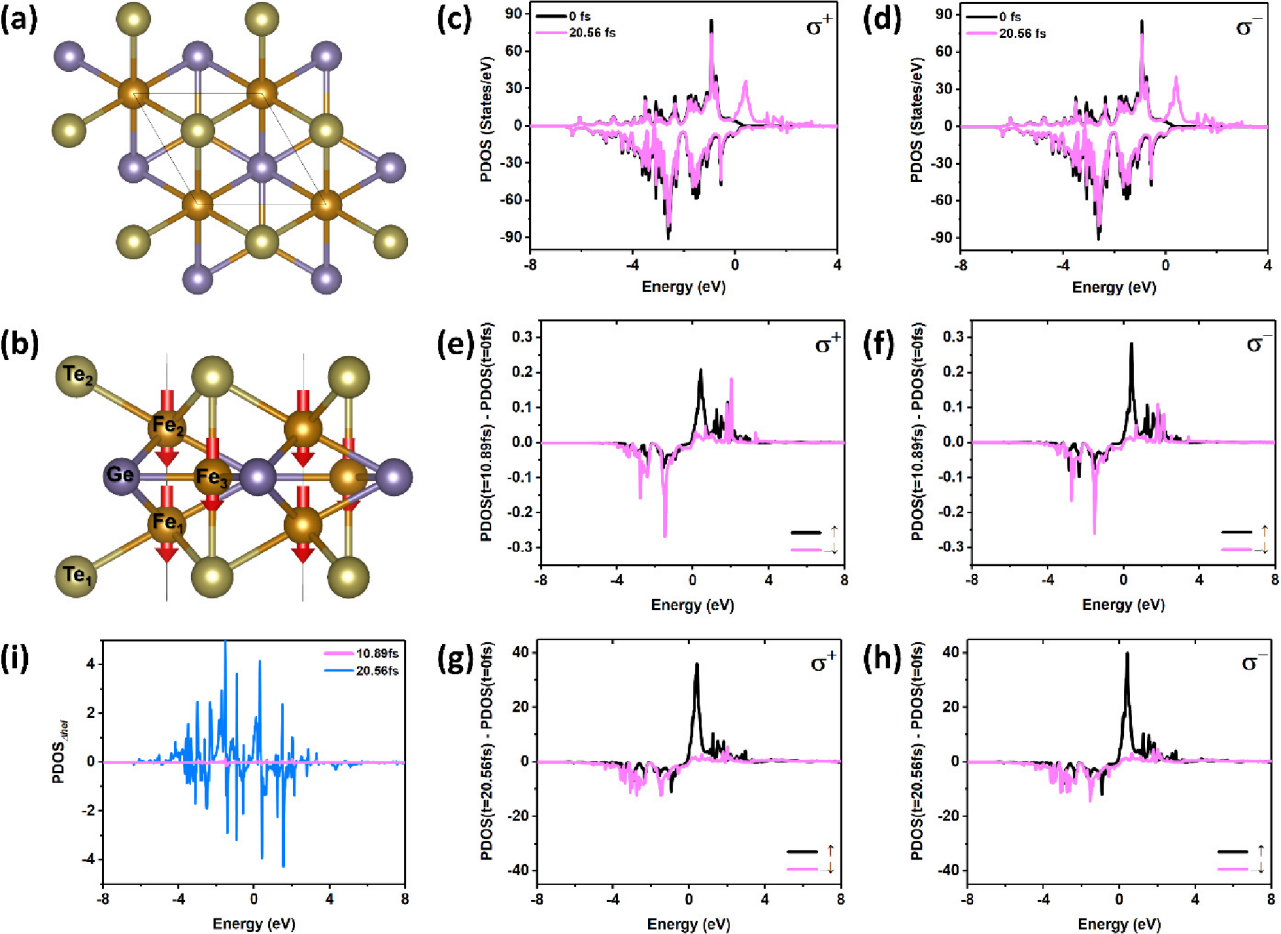
(a) Top view and (b) side view of the atomic configuration of FGT. The red arrows located on Fe atoms represent the spin. The time-resolved partial density of states (PDOS) of Fe_1_ at 0 and 20.56 fs by (c) σ^+^ and (d) σ^–^ polarized lasers. (e–h) Variation of the time-resolved PDOS of Fe_1_, PDOS(*t*) – PDOS(*t* = 0 fs), *t* = 10.89 and 20.56 fs by σ^+^ and σ^–^ polarized lasers. ↑ and ↓ correspond to the majority and minority, respectively. To improve the readability, the ↓ curves are multiplied by −1. (i) Difference of the helicity-dependent PDOS of Fe_1_ at 10.89 and 20.56 fs by polarized lasers, PDOS_Δhel_(*t*) = PDOS^σ+^(*t*) – PDOS^σ–^(*t*).

The previous work demonstrated that itinerant magnets of Fe atoms in FGT can be demagnetized in femtoseconds and excited by linear laser pulses.^45^ However, the spin dynamics of 2D magnetic materials manipulated by circularly left (σ^+^) and right (σ^–^) polarized lasers are rarely studied.^36^ Therefore, we first simulated the spin dynamics of each atom in FGT (Figure S1) excited by circularly left (σ^+^) and right (σ^–^) polarized lasers at different frequencies (*f*) (Figure S2), and the corresponding parameters of circularly polarized lasers are given in Table S1. The simulations show the strong out-of-plane (*M*_*z*_) demagnetization processes of Fe atoms, while Ge and Te atoms gain a small amount of spin (Figure S1). To further distinguish the difference of demagnetization in FGT between excited by σ^+^ and σ^–^ polarized lasers, we have defined the helicity-dependent (HD) and helicity-independent (HI) dynamics of Fe_1_ in FGT (Figure S3). The magnetic moment of Fe_1_ decomposes into a HI and a HD part as *M*_*z*_^σ^±^^(*t*) = *M*_*HI*_(*t*) ± *M*_*HD*_(*t*), wherein the HI part is written as  and the HD part is written as . Thus, the HI part is an average of the demagnetization, as in linear light excitation,^45^ and the HD part is the extra part, which is the helicity-dependent change in the demagnetization excited by circularly polarized lasers.

To understand the mechanism of the magnetization response of FGT excited by σ^+^ and σ^–^ polarized lasers, we analyzed the time-resolved electronic and magnetic properties. Figure 1c and 1d illustrates the time-dependent partial density of states (PDOS) of Fe_1_ in the case of σ^+^ and σ^–^ polarized lasers with a fixed fluence^47^ (*F* = 7.06 mJ/cm^2^). To clearly observe the difference in the time-dependent PDOS of Fe_1_, we also plot the changes in PDOS, i.e., PDOS(*t*) – PDOS(*t* = 0 fs), for σ^+^ and σ^–^ polarized lasers, as shown in Figure 1e–h. The change in PDOS of Fe_1_ in the [10.89, 20.56] fs time window is approximately a hundred times that in the [0, 10.89] fs time window. Therefore, *M*_*z*_ of Fe_1_ exhibits a strong demagnetization process in the [10.89, 20.56] fs time window due to the OISTR effect, and a similar case also happens to the Fe_3_ atom (Figure S4a). The change of occupied/unoccupied orbitals of Te_1_/Ge on majority/minority states at 20.56 fs is shown in Figure S4b,c. Moreover, we plotted PDOS_Δhel_(*t*) = PDOS^σ+^(*t*) – PDOS^σ–^(*t*) to confirm the contribution of helicity-dependent demagnetization of *M*_*z*_ of Fe_1_. The remarkable PDOS_Δhel_(*t*) of Fe_1_ during the [−6, 6] eV energy window is shown at 10.89 and 20.56 fs (Figure 1i). In fact, these peaks reveal the presence of 3d states of Fe_1_, which usually follow more helicity-dependent selection rules.^35^

To further analyze the spin transfer dynamics in FGT, we examined the time-dependent occupation changes (Δ*n*(*t*)) in Fe_1_, Fe_3_, Ge, and Te_1_ atoms, i.e., Δ*n*(*t*) = *n*(*t*) – *n*(0), as shown in Figure S5. We also observed that the majority/minority electrons of Fe_1_/Fe_3_ are rapidly gained/lost in the [10.89, 20.56] fs time window, corresponding to the decrease in the magnetic moment. The opposite is true for Te_1_/Ge. The time-resolved occupation dynamics show that the photoinduced spin-selective charge flows from Fe to Te_1_/Ge; i.e., the OISTR mechanism dominates the direct spin transfer from Fe to Te_1_/Ge in the [10.89, 20.56] fs time window. In addition, we also examined helicity-dependent occupation changes (*n*_Δhel_(*t*)) in Fe_1_, Fe_3_, Ge, and Te_1_ atoms, i.e., *n*_Δhel_ = *n*^σ+^(*t*) – *n*^σ–^(*t*). The results show that the change in the helicity-dependent majority/minority electrons is small but induces an oscillation process in the occupation dynamics due to the linear response of the optical helicity.

The above results show the rapid demagnetization of FGT in the [10.89, 20.56] fs time window, where the demagnetization response of FGT is mainly derived from the change of their out-of-plane magnetism by the OISTR mechanism. However, the response of the in-plane magnetism (*M*_*x*/*y*_) is not clear, especially in the [0, 10.89] fs time window. The dynamics of the 3-dimensional magnetization is even more complicated than its *z*-component, because the transverse components of *M*_*x*/*y*_ also change rapidly depending on the polarized lasers.^47^ Therefore, the next step would be to further investigate the in-plane spin dynamics.

We simulated the three-dimensional magnetization response of FGT to σ^+^ and σ^–^ polarized lasers in 30 fs with *F* = 7.06 mJ/cm^2^ and the full-width at half-maximum (fwhm) of 9.68 fs. The case of introducing the σ^+^ polarized laser illustrates the *M*_*z*_ of the Fe_1_ atom quickly reducing and the clockwise rotation in the *xy* plane (Figure 2a), while the case of introducing the σ^–^ polarized laser illustrates the *M*_*z*_ quickly reducing and the anticlockwise rotation in the *xy* plane (Figure 2b). In addition, we have also calculated the three-dimensional magnetization response of other atoms in FGT, in which the helicity-dependent magnetization processes of Te_1_ atoms are also shown in 30 fs (Figure S6a). However, Fe_3_ and Ge atoms in the middle layer of FGT do not show the helicity-dependent magnetization processes (Figure S6b and c), which might be due to the fact that the covalent interaction between the 3d orbitals of Fe_3_ and Ge (see the electron localization function of FGT in Figure S7) is much stronger than the light–spin coherence; i.e., the helicity-dependent spin dynamics only exhibits the demagnetization processes of Fe_1_ and Te_1_ due to more relatively itinerant d electrons in Fe_1_ and Te_1_ than that in Fe_3_ and Ge. The results show that σ^+^ and σ^–^ polarized lasers can induce the helicity-dependent demagnetization processes in FGT, i.e., the in-plane spin response. In fact, this in-plane spin dynamics of Fe_1_ and Te_1_ is derived from their angular momentum transfer between the polarized fields of the laser pulses, orbital and spin on the attosecond time scale.^36,47^ Therefore, the orbital and spin angular momentum in FGT must be further distinguished.

**Figure 2:**
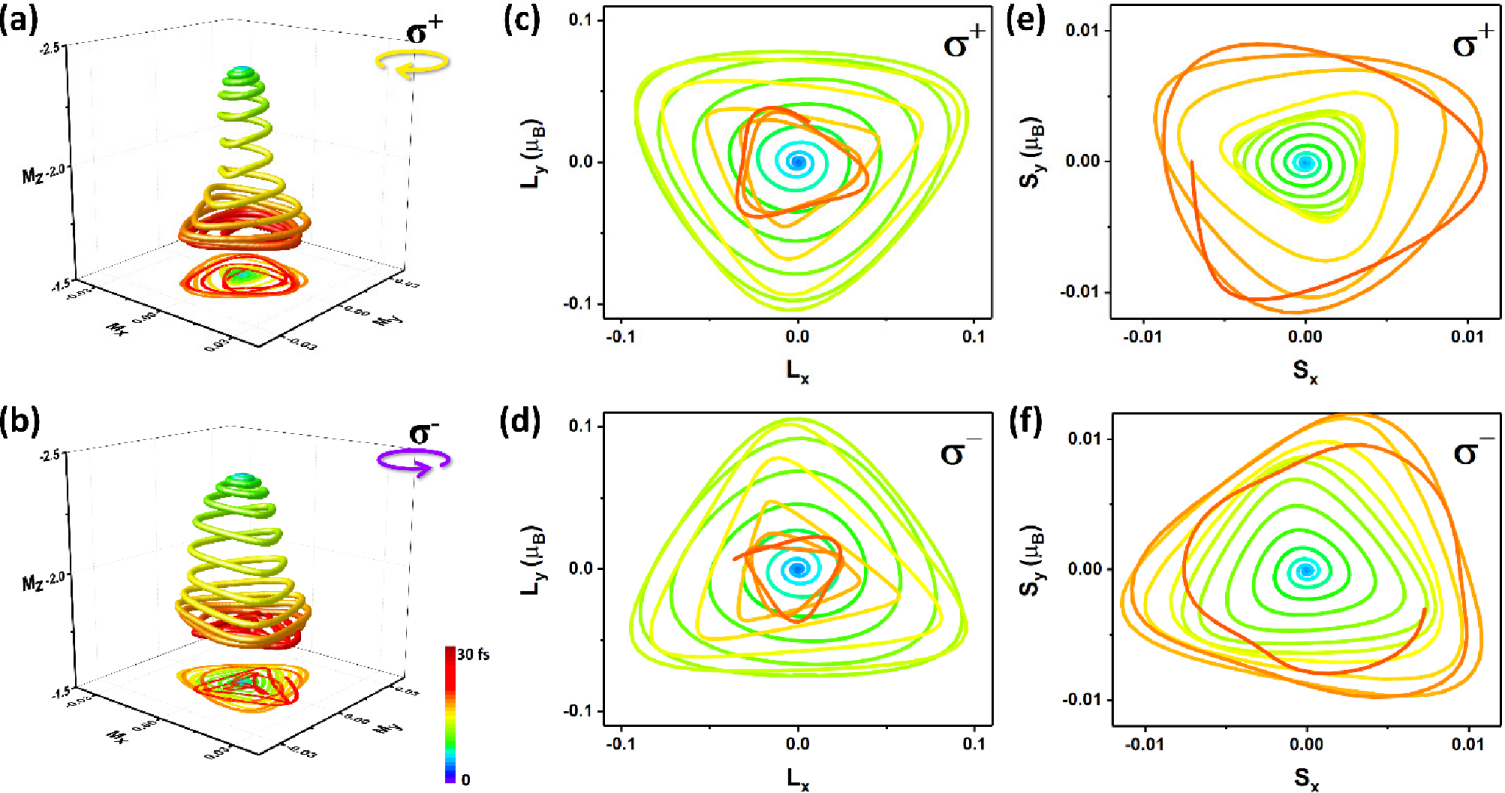
Three-dimensional demagnetization dynamics of the Fe_1_ atom in FGT excited by circularly (a) left (σ^+^) and (b) right (σ^–^) polarized lasers. The clockwise and anticlockwise rotations of (c and d) the OAMin the *xy* plane (*L*_*x*_ and *L*_*y*_) and (e and f) the SAM in the *xy* plane (*S*_*x*_ and *S*_*y*_) are shown excited by circularly σ^+^ and σ^–^ polarized laser pulses, respectively. The color bar is the time scale from 0 to 30 fs.

The light–matter interactions imply that the loss of the magnetic moments results in a transfer between the angular momentum of polarized fields of laser pulses, the orbital angular momentum (OAM, **L**) and the spin angular momentum (SAM, **S**), where **L** and **S** are their expectation values and are defined in the Supporting Information. Taking the Fe_1_ atom as an example, we can trigger different **L** and **S** in Fe_1_ of FGT excited by circularly polarized laser pulses. Our simulations show that the OAM in the *xy* plane (*L*_*x*_ and *L*_*y*_) first increases and then decreases (Figure 2c and d). Moreover, the clockwise and anticlockwise rotations of the OAM in the *xy* plane are observed by circularly induced σ^+^ and σ^–^ polarized lasers, respectively. The SAM in the *xy* plane (*S*_*x*_ and *S*_*y*_) also exhibits these clockwise and anticlockwise rotations (Figure 2e and f). Therefore, the in-plane motions of the OAM and SAM are similar to circularly polarized laser pulses, indicating the linear response between the angular momentum of polarized fields of laser pulses, OAM and SAM, through the optical helicity.

To understand the angular momentum transfer from the polarized fields of laser pulses to the OAM, we further analyzed the in-plane response of optical helicity between the polarized fields of laser pulses and *L*_*x*/*y*_. Figure 3 shows the off-resonance (left panels) and on-resonance (right panels) cases and exhibits several features of the polarized fields of laser pulses and the linear response of *L*_*x*/*y*_ (see the blue dotted lines) excited by circularly σ^+^ and σ^–^ polarized lasers. The periodic response of the off-resonance and on-resonance cases evolves intrinsically on very ultrafast time scales of ∼600 attoseconds (inset of Figure 3b) from one extremum of *L*_*x*/*y*_ to the next. Furthermore, we tested different lifetime responses of *L*_*x*/*y*_ excited by circularly σ^+^ and σ^–^ polarized lasers with different fwhm values (Figure S8). The simulations show that the lifetime of the linear response of the OAM can remain longer excited by circularly σ^+^ and σ^–^ polarized lasers with a wider fwhm.

**Figure 3:**
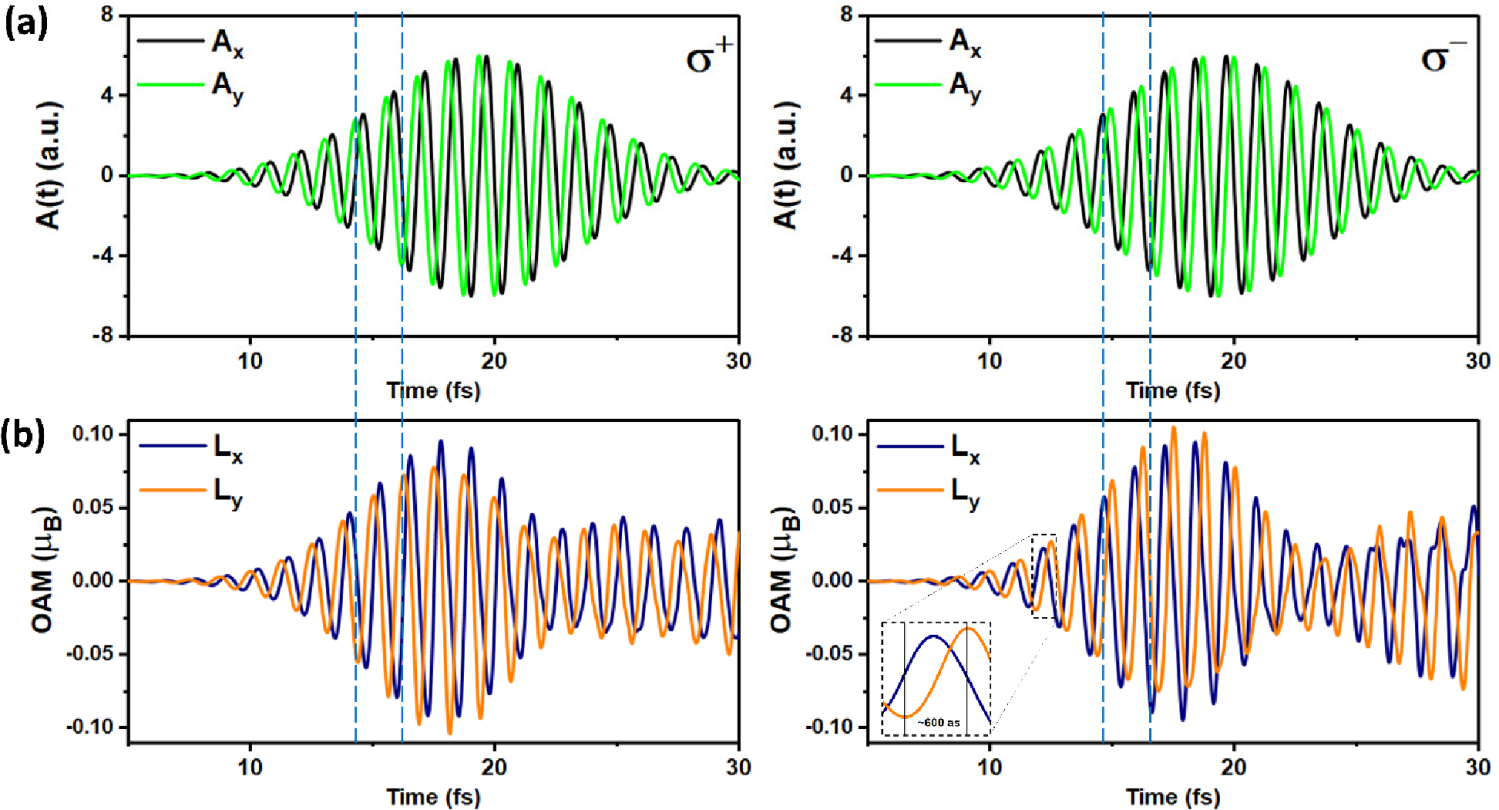
The panels on the left and right columns represent the cases of induced circularly σ^+^ and σ^–^ polarized lasers, respectively. From top to bottom, the panels show (a) the time dependence of the amplitude vector potential in the *xy* plane (*A*_*x*_ and *A*_*y*_) of laser pulses and (b) the OAM. The periodically linear response of *L*_*y*_ is ∼600 attoseconds, as shown in the inset.

Efficient transfer between orbital and spin magnetic moments in condensed matter can occur at the subfemtosecond time scale through the SOC effect.^14^ Therefore, we now investigate the angular momentum transfer between the orbital and spin in FGT after the ultrafast response of the orbital with circularly polarized lasers. Such behavior parallels that of classical gyroscope precession, where a decrease in precession frequency leads to an increase in precession angle. Therefore, the precession angular of **L** (θ_**L**_) first increases to 90°, resulting in an unstable precession and then a flip of **L**. After 17.5 fs, **L** still obtains the angular momentum from the laser, resulting in the precession angle of **L** (θ_**L**_) decreasing (Figure 4a). In the case of the Fe_1_ atom excited by the circularly σ^+^ polarized laser pulse, **L** remains in the −*z* direction, and there is no flip. Moreover, the precession angles of **S** (θ_**S**_) are less than 1 degree (Figure 4b). Interestingly, the θ_**L**_ is much larger than that of θ_**S**_, indicating that the precession of **L** is much slower than that of **S**, like a classical gyroscope precession. The above results for **L** and **S** in FGT are similar to previous experimental and theoretical works on metals using linearly polarized lasers.^14,48^ The simulations show that the estimated change of the OAM (∼8 fs) is earlier than that of the SAM (∼10.50 fs). The hysteretic transfer between **L** and **S** could be mediated by the SOC effect.^34^ Therefore, the linear response of the optical helicity for **L** is faster than that for **S**, suggesting that the angular momentum of the polarized fields of the laser pulses is transferred first to **L** and then to **S**.

**Figure 4:**
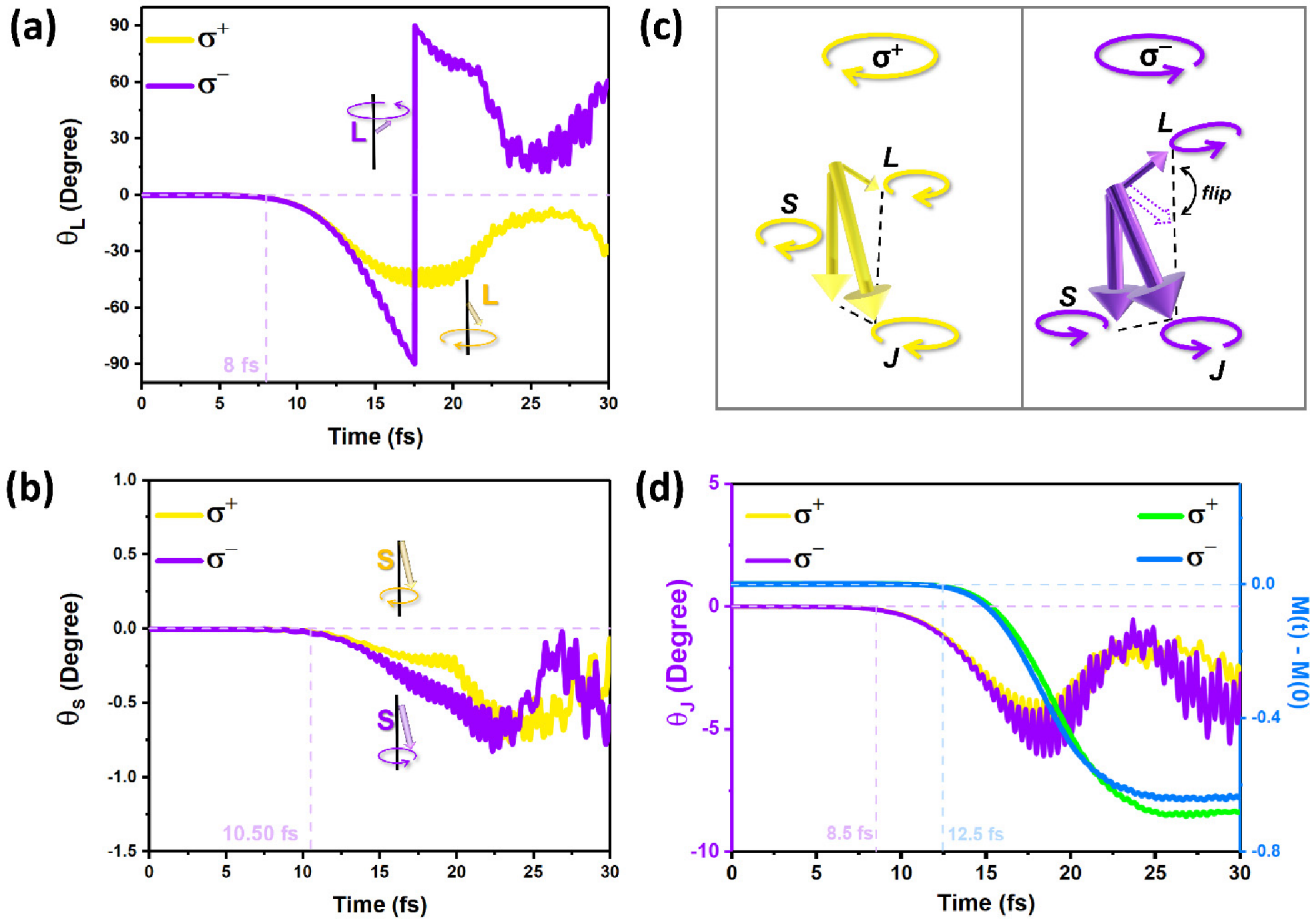
(a) The time-dependent dynamics of the precession angle of **L** (θ_**L**_). (b) The time-dependent dynamics of the precession angle of **S** (θ_**S**_). The *z* direction is defined as the positive direction. The estimated initial times of the significant change in θ_**L**_ and θ_**S**_ are marked with dotted lines, and the precessional motion of **L** and **S** is shown in the illustration. (c) The schematic diagram for the evolution of **J**, **L**, and **S** excited by circularly left (σ^+^) and right (σ^–^) polarized laser pulses. (d) The time-dependent dynamics of the precession angle of **J** (θ_**J**_) and the total magnetism dynamics of Fe_1_ atom, Δ*M* = *M*(*t*) – *M*(0). The estimated initial times of the significant changes in θ_**J**_ and Δ*M* are marked with dotted lines.

Based on the above analysis, we plotted the schematic diagram for the total angular momentum **J** = **L** + **S**, with **L** and **S** excited by circularly polarized laser pulses (Figure 4c). Notably, **L** exhibits an instantaneous flip (in the case of a circularly σ^–^ polarized laser pulse), but **S** is 10 times bigger than **L**. Consequently, θ_**L**_ is unlikely to substantially influence the precession angle of **J** (θ_**J**_). To further distinguish the evolutions of **J** and demagnetization in Fe_1_ of FGT, we plotted its θ_**J**_ and the change of its total magnetism dynamics of Δ*M* = *M*(*t*) – *M*(0) excited by circularly σ^+^ and σ^–^ polarized lasers (Figure 4d), respectively. There is an estimated delay of ∼4 fs between the significant change in θ_**J**_ and that in Δ*M*, indicating that the angular momentum transfer between the polarized fields of laser pulses and **J** by the linear response is faster than the demagnetization by the OISTR effect in FGT. Therefore, angular momentum manipulation using circularly polarized lasers could be the fastest methods currently available in 2D magnets^36^ for ultrafast spintronics.

To further confirm the general regularity of the orbital and spin dynamics in 2D ferromagnets, we simulated the demagnetization of Cr atoms in two FM semiconductors of Cr_2_Ge_2_Te_6_^49^ (CGT) (Figure S9a) and CrI_3_^50^ (Figure S9b), which have been experimentally prepared and exhibit the controllable magnetic properties including magnetic states and magnetic phase transitions by applying external fields.^51−56^ The simulations show the clockwise and anticlockwise rotation demagnetization of Cr atoms in CGT (Figure S9c,d) and CrI_3_ (Figure S9e,f) by induced σ^+^ and σ^–^ polarized lasers, respectively. Moreover, we also find the periodic response between circularly polarized lasers and the OAM of Cr sublayers in CGT (Figure S10) and CrI_3_ (Figure S11). Therefore, the ultrafast orbital and spin dynamics in CGT and CrI_3_ are similar to those in FGT. Furthermore, the recent work^47^ shows that an increase in SOC (scaled by factors of 1.5 and 2.0) for the in-plane dynamics of SAM and OAM in Co ferromagnets leads to an increase in SAM but no change in OAM. Consequently, the SAM dynamics in FGT, CGT, and CrI_3_ are different due to the different chemical environment of the magnetic atoms, resulting in different SOC strengths. The above results indicate that the manipulation of the orbital and spin angular momentum using circularly polarized lasers could be common and easy to achieve in 2D ferromagnets. The study of orbital and spin dynamics in 2D magnetic materials will open up new perspectives in ultrafast physics in the field of femtomagnetism and attomagnetism.^36,47^

The possibility of coherent control of spin dynamics on subfemtosecond time scales, i.e., the new field of attomagnetism, has been proposed.^1,36^ We predict the optical-helicity-dependent orbital and spin dynamics in FGT, where the linear response occurs on attosecond time scales and is mediated by cascaded light–orbit–spin interactions. This suggests that manipulation of orbital and spin angular momentum with attosecond lasers could be a potential application in the field of attomagnetism for spintronics and orbitronics.^57^ As an emerging and active field, attomagnetism in condensed matter physics will continue to provide new scientific insights and inspiration for new scientific frontiers in ultrafast materials science. Such studies^58^ will also pave the way for advanced science and technology in future attosecond spintronics. In addition, our simulations focused specifically on early spin dynamics, thus limiting the time scale to within 100 fs. However, on the 50–100 fs time scale, electron–phonon coupling will play a crucial role in influencing the precession of the SAM and the OAM, leading to a complex angular momentum transfer involving phonons. Recently, the induced coherent phonon dynamics of FGT is strongly coupled to the spin dynamics in the 200–800 fs range.^59^ In addition, circularly polarized phonons or chiral phonons have been observed in the demagnetization process^60^ due to angular momentum transfer from spin systems. Therefore, the angular momentum transfer between SAM/OAM dynamics and polarized phonons is an interesting and open problem that deserves further investigation.

In conclusion, we demonstrate that, for FGT, (i) the helicity-dependent orbital and spin dynamics show at a few femtoseconds excited by circularly polarized laser pulses. (ii) The in-plane orbital and spin dynamics by linear response are faster than their out-of-plane dynamics by the OISTR, and the periodic response of the angular momentum excited by circularly polarized laser pulses occurs in ∼600 attoseconds. And (iii) the microscopic mechanism of the helicity-dependent spin dynamics is the ultrafast transfer between the angular momentum of polarized fields, the OAM, and the SAM through the optical linear response and the SOC effect. These results pave the way for manipulating the angular momentum by circularly polarized lasers in 2D magnets. The underlying mechanism of orbital and spin dynamics suggests that such 2D magnetic materials will provide a great platform for manipulating the light–orbital–spin interaction for ultrafast spintronics in the field of femtomagnetism and attomagnetism.

## Supporting Information Available

Methods and computational details, demagnetization of FGT by polarized lasers with different frequencies, circularly polarized fields with different frequencies, HI and HD spin dynamics, the time-resolved PDOS of Te_1_, Fe_3_, and Ge and their variation, the time-dependent change of occupations, 3-dimensional demagnetization dynamics of Te_1_, Fe_3_, and Ge, electron localization function of FGT, OAM response of Fe_1_ in FGT by polarized lasers with different fwhm, 3-dimensional demagnetization of the Cr atoms in CGT and in CrI_3_, OAM response of the Cr atom in CGT, OAM response of the Cr atom in CrI_3_, and a table of the corresponding laser parameters (PDF)
