# Supplementary material for: Optical-Helicity-Dependent Orbital and Spin Dynamics in Two-Dimensional Ferromagnets
Source: J Phys Chem Lett. 2024 May 29;15(22):5939–46. doi: 10.1021/acs.jpclett.4c01152 (PMC11163468; doi:10.1021/acs.jpclett.4c01152)
Supplement: Supplementary file 1 — jz4c01152_si_001.pdf [file jz4c01152_si_001.pdf]

Supporting Information

Optically Helicity-Dependent Orbital and Spin Dynamics in Two-Dimensional Ferromagnets

Shuo Li [1*], Ran Wang [1], Thomas Frauenheim [1, 2], Junjie He [3*]

[1] Institute for Advanced Study, Chengdu University, Chengdu 610106, China

[2] School of Science, Constructor University, Bremen 28759, Germany

[3] Department of Physical and Macromolecular Chemistry, Faculty of Science, Charles University, Prague 12843, Czech Republic

* Corresponding authors.

Email: shuoli.phd@gmail.com, junjie.he@natur.cuni.cz

Additional details of simulations

Methods and computational details

To identify the spin dynamics of FGT under the influence of ultrafast laser pulses, we performed real-time time-dependent density functional theory (rt-TDDFT) calculations. The time evolving state functions (ψ) were calculated by solving the time dependent Kohn-Sham (KS) equation as follows:

$$i\frac{\partial\psi_j(\mathbf{r},t)}{\partial t} = \left[\frac{1}{2}\left(-i\nabla + \frac{1}{c}\mathbf{A_{ext}}(t)\right)^2 + v_s(\mathbf{r},t) + \frac{1}{2c}\boldsymbol{\sigma}\cdot\mathbf{B_s}(\mathbf{r},t) + \frac{1}{4c^2}\boldsymbol{\sigma}\cdot(\nabla v_s(\mathbf{r},t)\times(-i\nabla))\right]\psi_j(\mathbf{r},t) \quad (1)$$

where $\mathbf{A_{ext}}(t)$ and σ represent vector potential and Pauli matrices. The KS effective potential $v_s(\mathbf{r},t) = v_{ext}(\mathbf{r},t) + v_H(\mathbf{r},t) + v_{xc}(\mathbf{r},t)$ can be decomposed into the external potential v_{ext}, the classical Hartree potential v_H, and the exchange-correlation (XC) potential v_{xc}, respectively. The KS magnetic field can be written as $\mathbf{B_s}(\mathbf{r},t) = \mathbf{B_{ext}}(\mathbf{r},t) + \mathbf{B_{xc}}(\mathbf{r},t)$, where $\mathbf{B_{ext}}$ and $\mathbf{B_{xc}}$ represent the magnetic field of the applied laser pulse with an additional magnetic field and XC magnetic field, respectively. The last term in Eq. (1) stands for the SOC effect. The magnetization density can be calculated as $\boldsymbol{m}(\mathbf{r},t) = \sum_j \psi_j^*(\mathbf{r},t)\boldsymbol{\sigma}\psi_j(\mathbf{r},t)$ and the integral of this vector field over the unit cell leads to the spin angular momentum (**S**).

In general, the orbital angular momentum operator $\mathbf{L} = \mathbf{r} \times \mathbf{p}$ cannot be strictly defined because the position operator **r** cannot be well defined in a periodic solid[1]. However, if the angular momentum arises from orbitals localized within the muffin tin (i.e., a sphere around the point nuclei), which should be as large as possible. This approach can still be employed: one sets $\mathbf{r} = 0$ as the center of the muffin tin and performs the integral of the **L** expectation value only within this sphere. The basic assumption for the transient case is that the current loops induced by laser pulses are predominantly contained within the muffin tin[2]. The low fluence of laser pulses is performed for the validity of the orbital angular momentum[3].

The interactions of electrons with the laser are described in the velocity gauge, and the time-dependent vector potential **A**(t) is from $\boldsymbol{E}(t) = -\frac{1}{c}\frac{\partial \boldsymbol{A}(t)}{\partial t}$, where the 1/c is a factor (see details in the ELK code). The homogeneous circularly polarized fields are defined to trigger the generation of an orbital angular momentum in the system. The **A**(t) of homogeneous electric field can be written as $\boldsymbol{A} = (A_x, A_y, 0)$,

$$A_x(t) = \begin{cases} A_0 \, cos(\omega t) sin\left(\frac{\pi t}{T}\right), & 0 \le t \le T \\ 0, & otherwise \end{cases} \tag{2}$$

$$A_y(t) = \begin{cases} A_0 \, cos(\omega t - \varphi) sin\left(\frac{\pi t}{T}\right), & 0 \le t \le T \\ 0, & otherwise \end{cases} \tag{3}$$

where φ is the polarization angle and φ = ±90° are used for circularly σ+ and σ− polarized laser pulses. The second sinusoidal term of Eq. (2) and Eq. (3) constitutes the temporal envelope of the pulse, in which T is a duration. In addition, corresponding parameters of laser pulses are shown in Table S1.

In the DFT calculations, the Brillouin zone was represented by a Γ Monkhorst-Pack grids of 11 × 11 × 1 for structure relaxation and electronic structures. An energy cut-off of 500 eV was used to determine the self-consistent charge density for the plane wave basis sets. The structures were fully optimized until the maximum force on atoms was lower than 0.01 eV/Å and the total energy variation was lower than 1.0×10^{-5} eV. A vacuum of approximately 12 Å was added in the perpendicular direction to the slab model.

In the rt-TDDFT simulations, we only considered the spin polarized electron dynamics based on the Born-Oppenheimer approximation. The electron-phonon coupling effects are not taken into account. Photoinduced dynamics calculations were made using a fully non-collinear version of rt-TDDFT and a full-potential augmented plane-wave ELK code.[4] A regular mesh in a k-space of 6 × 6 × 1, a smearing width of 0.027 eV, and a time step of Δt = 0.1 a.u. were used to simulate excited dynamics. The laser pulses that were used in the present study were linearly polarized (in-plane polarization) at a selected frequency. All calculations were performed using adiabatic local spin density approximations (ALSDA)[5].

Notes on parameters of laser pulses for rt-TDDFT simulations

Employing state-of-the-art rt-TDDFT we show that spin-orbit coupling at femtosecond timescales drives a transfer among the polarized fields of laser pulses, the orbital angular momentum and the spin angular momentum. The rate of change of orbital and spin momentum have predominantly extrinsic (the pulse duration) and intrinsic (the spin-orbit coupling) timescale, respectively. In the rt-TDDFT simulations, the fluence of laser pulses are mainly determined by the related amplitude vector potential (A), frequency (f), and full width at half maximum (FWHM). Therefore, we showed the different laser pulses in Figure S3 and Figure S4, and their parameter details were summarized in Table S1. The FWHM in first four rows of this table are fixed for testing the influence of frequency, and the f in last four rows of this table are fixed for testing the influence of the orbital angular momentum lifetime of FWHM.

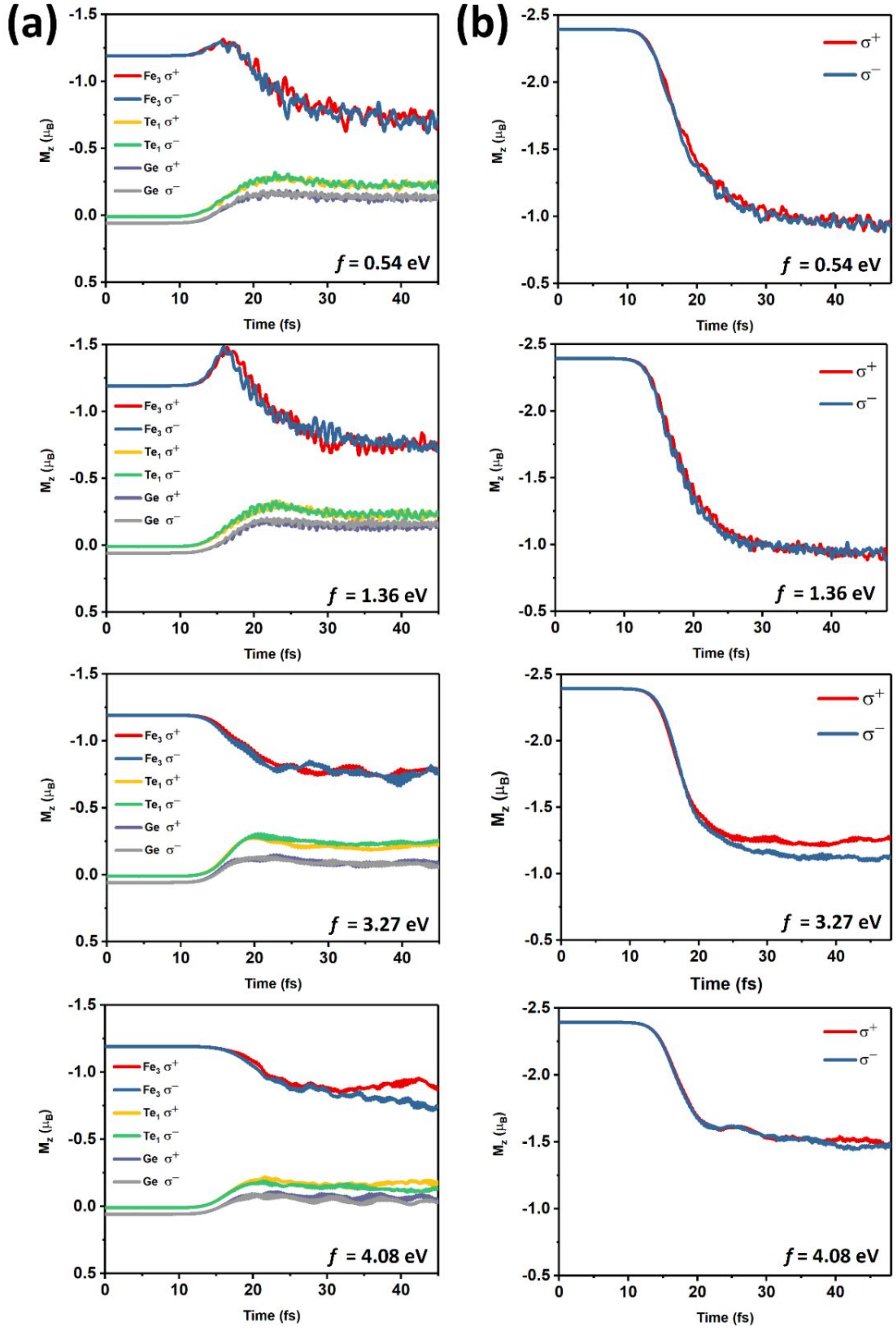

Figure S1 The demagnetization of M_z of (a) F_3, Te_1, Ge and (b) Fe_1 excited by circularly left (σ^+) and right (σ^-) polarized lasers with frequencies $f = 0.54$, 1.36, 3.27 and 4.08 eV.

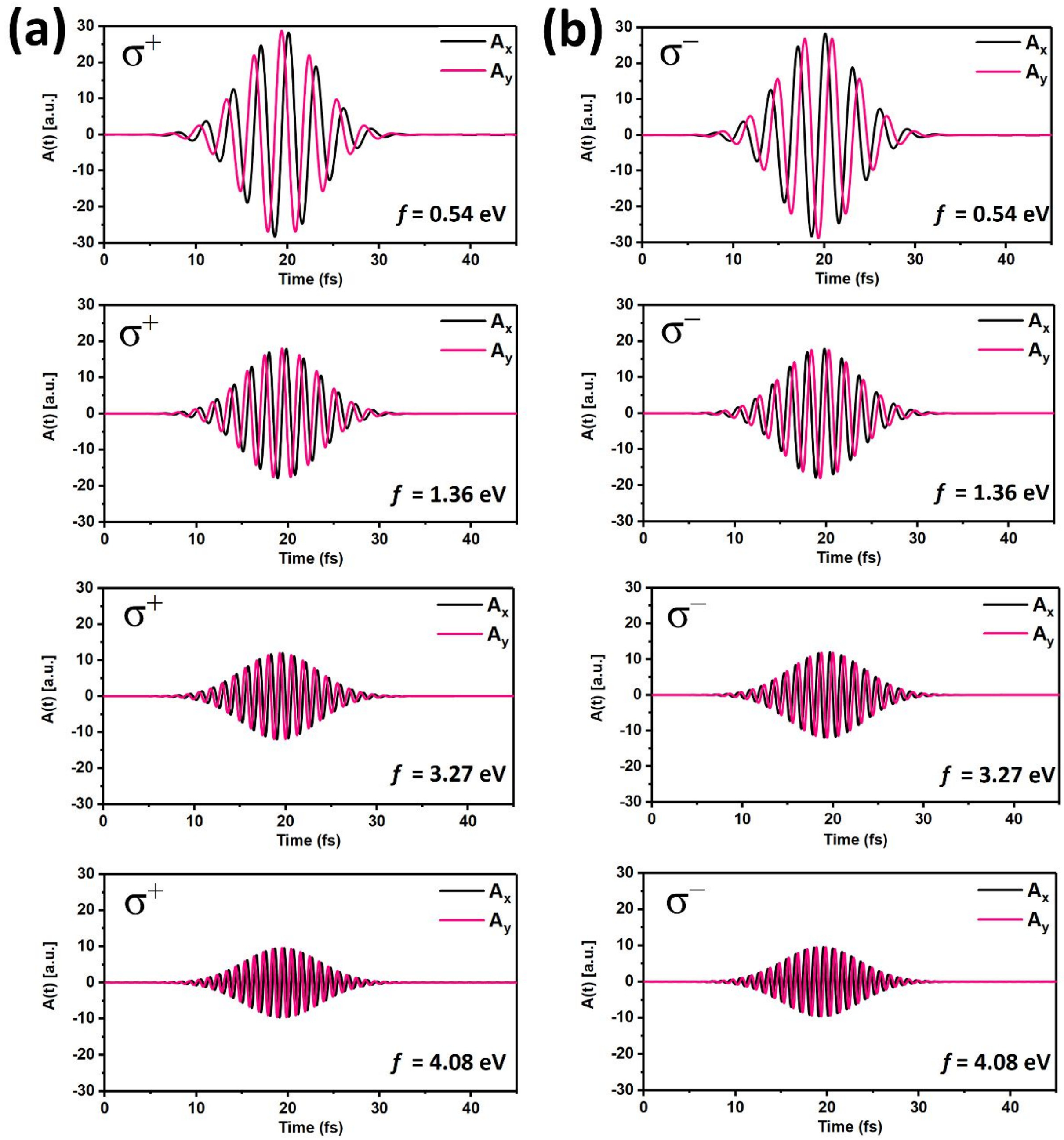

Figure S2 The circularly (a) left (σ^+) and (b) right (σ^-) polarized lasers are simulated by the homogeneous circularly polarized fields under frequencies *f* = 0.54, 1.36, 3.27 and 4.08 eV. The amplitude vector potential in xy plane (A_x and A_y) of the laser pulse is shown.

To highlight the helicity-dependent (HD) dynamics of Fe sublattices excited by circularly polarized lasers, we decompose magnetic moment in a helicity-independent (HI) and a HD part as $M_z^{\sigma^\pm}(t) = M_{HI}(t) \pm M_{HD}(t)$, in which the HI part writes as $M_{HI}(t) = \frac{1}{2}[M_z^{\sigma^+}(t) + M_z^{\sigma^-}(t)]$ and the HD part as $M_{HD}(t) = \frac{1}{2}\left[M_z^{\sigma^+}(t) - M_z^{\sigma^-}(t)\right]$.

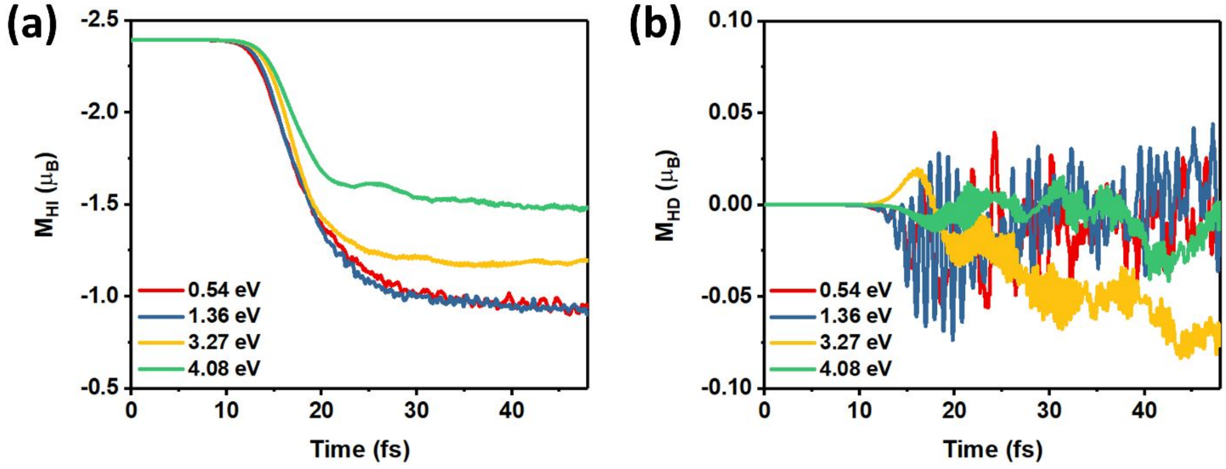

Figure S3 (a) The helicity-independent (HI) and (b) the helicity-dependent (HD) spin dynamics of Fe_1 in FGT under different frequencies.

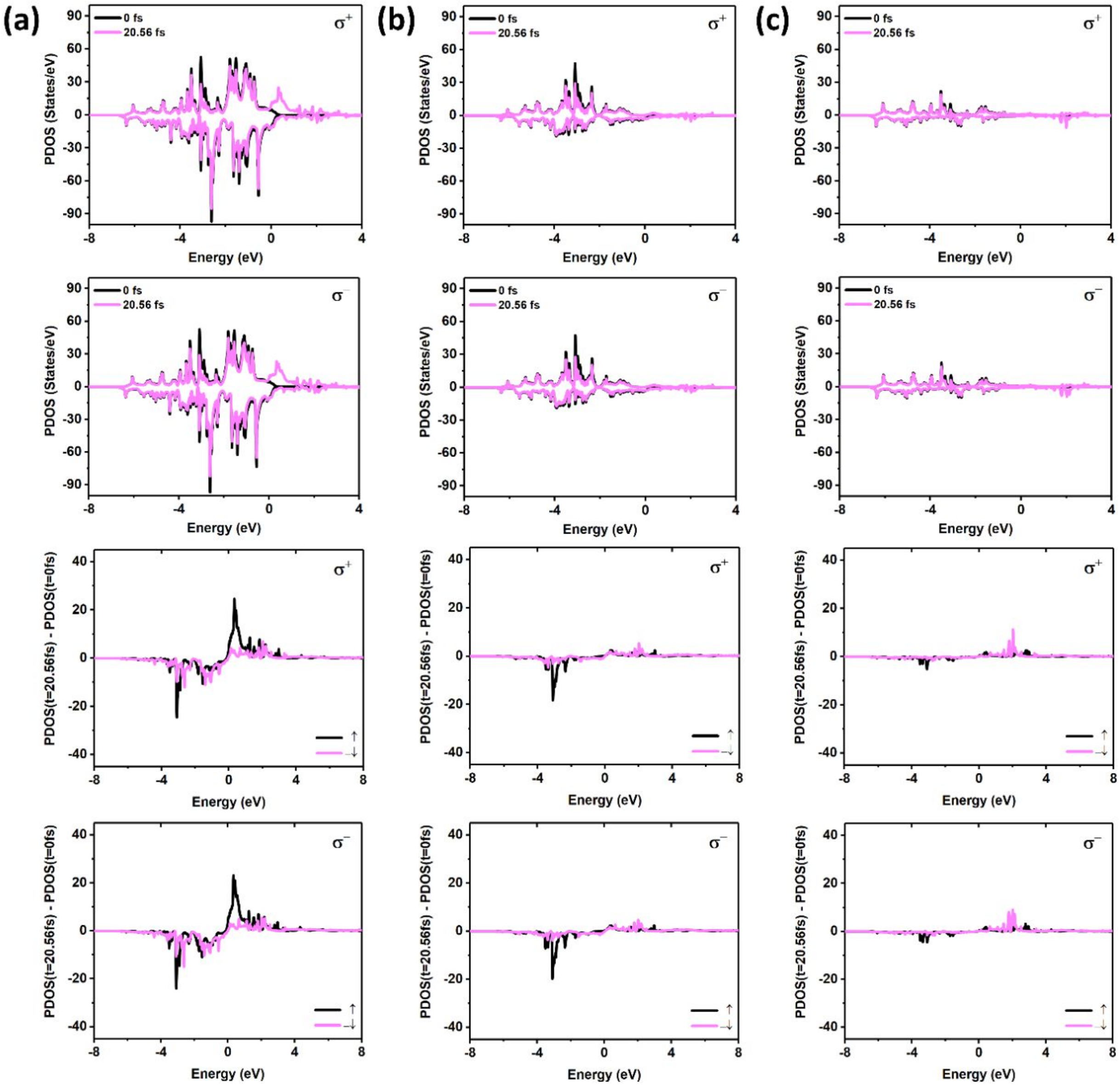

Figure S4 The time-resolved partial density of states (PDOS) and variation of the time-resolved PDOS for (a) Fe_3, (b) Te_1 and (c) Ge at 0 and 20.56 fs by σ^+ and σ^- polarized lasers. ↑ and ↓ correspond to the majority and minority, respectively. To improve readability, the ↓ curves are multiplied by −1.

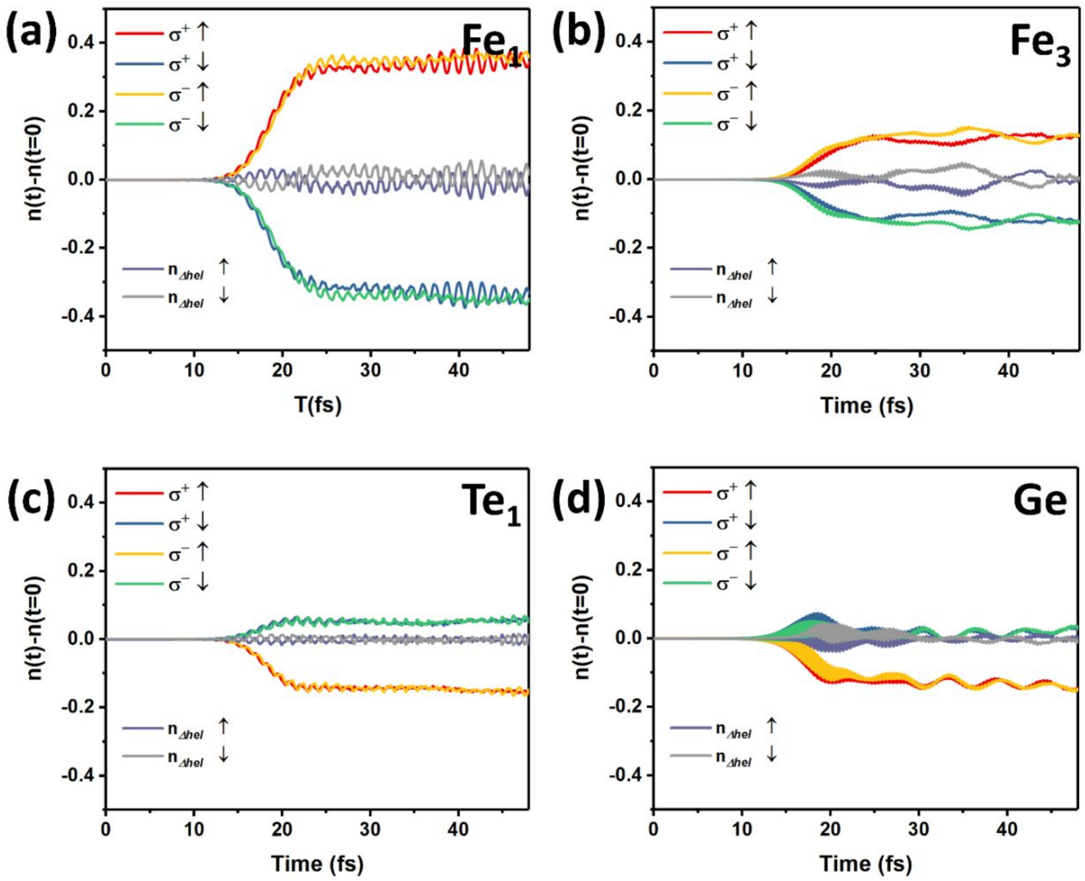

Figure S5 The time dependent change of majority, minority and helicity-dependent occupations as a function of time (in fs) of (a) Fe_1, (b) Fe_3, (c) Te_1 and (d) Ge atoms, which is defined as $\Delta n = n(t) - n(t=0)$.

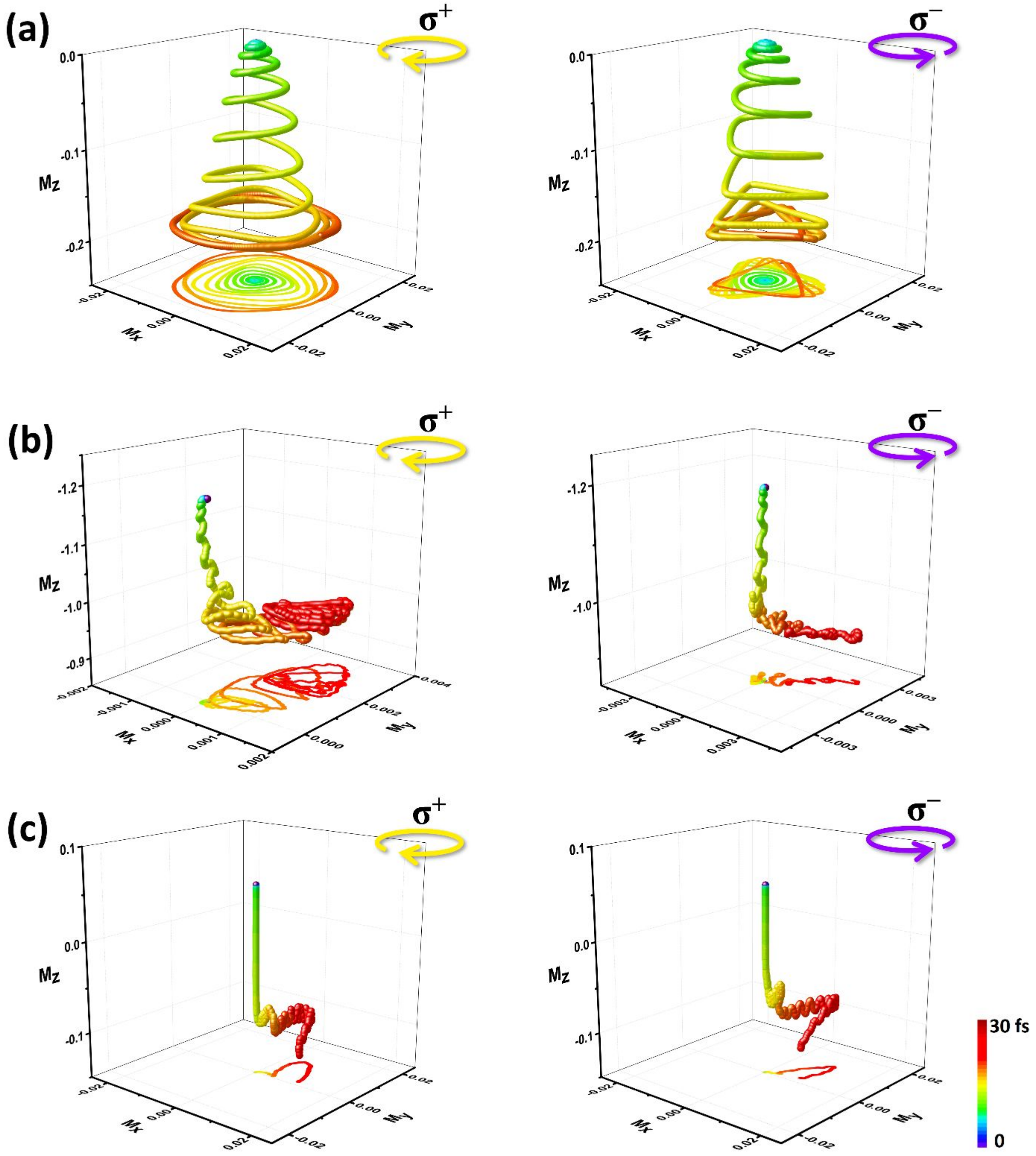

Figure S6 The 3-dimensional demagnetization dynamics of the (a)Te_1, (a)Fe_3 and (a)Ge atoms in FGT excited by circularly left (σ^+) and right (σ^-) polarized lasers. The color bar is the timescale from 0 to 30 fs.

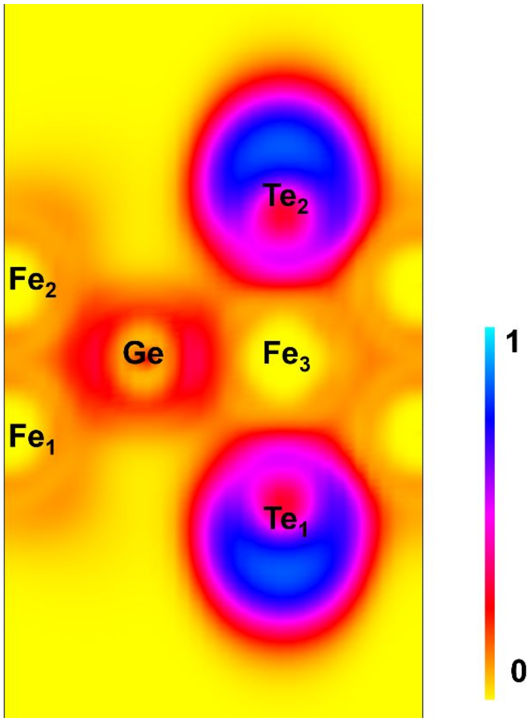

Figure S7 Electron localization function (ELF) maps of FGT. The ELF slice is along (110) surface. The unit of the color scale is "probability".

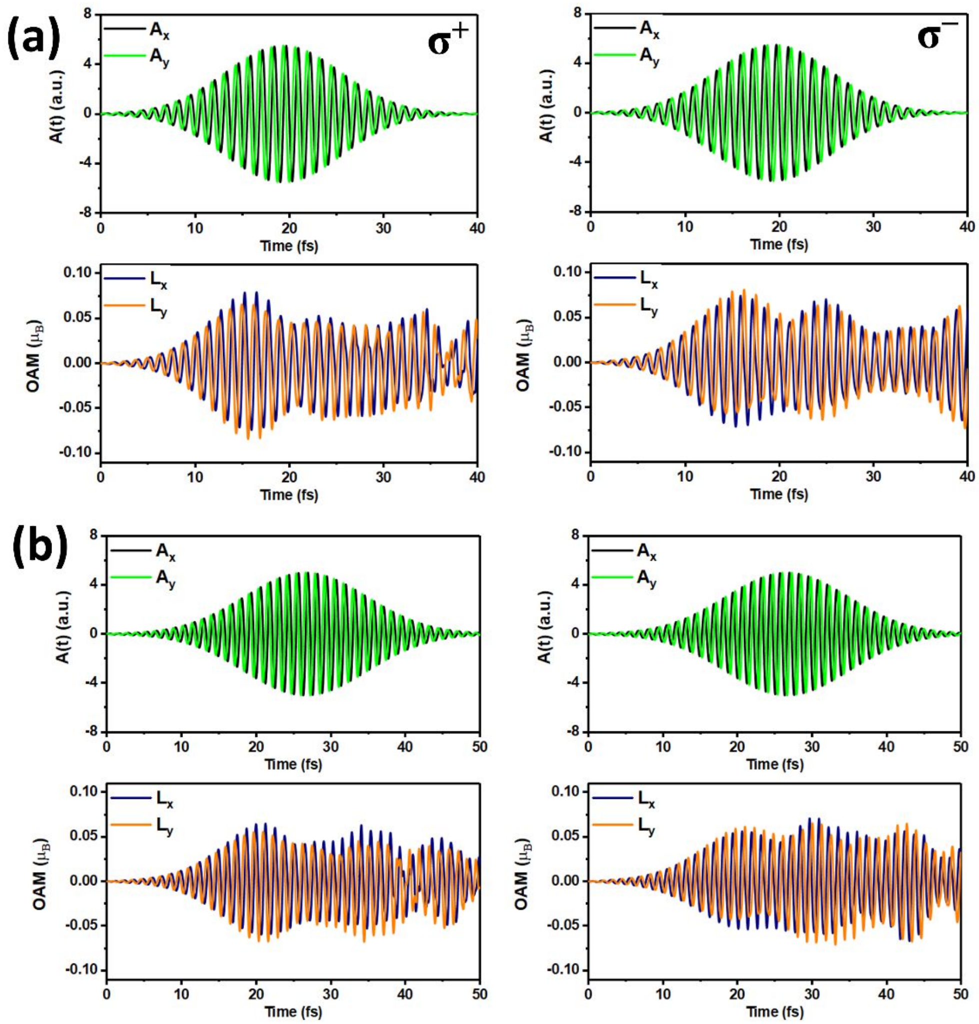

Figure S8 Panels on the left and right columns represent the cases of circularly σ^+ and σ^- polarized lasers induced, respectively. From top to bottom, the panels show the time dependence of the amplitude vector potential in xy plane (A_x and A_y) of laser pulses with the full width at half maximum FWHM = (a) 14.51 fs and (b) 19.35 fs and corresponding OAM.

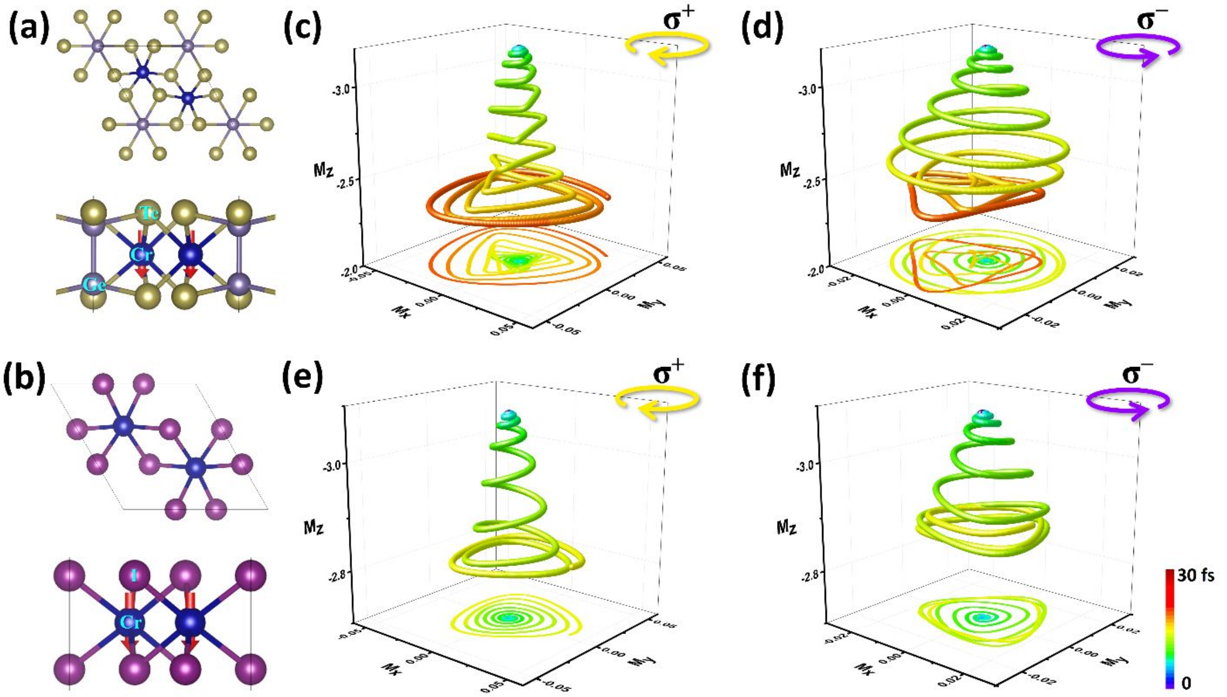

Figure S9 The atomic configuration of (a) CGT and (b) CrI_3. The red arrows located on Cr atoms represent the spin. The 3-dimensional demagnetization of the Cr atoms in CGT (c and d) and in CrI_3 (e and f) excited by circularly left (σ^+) and right (σ^-) polarized laser pulses, respectively. The color bar is the timescale from 0 to 30 fs.

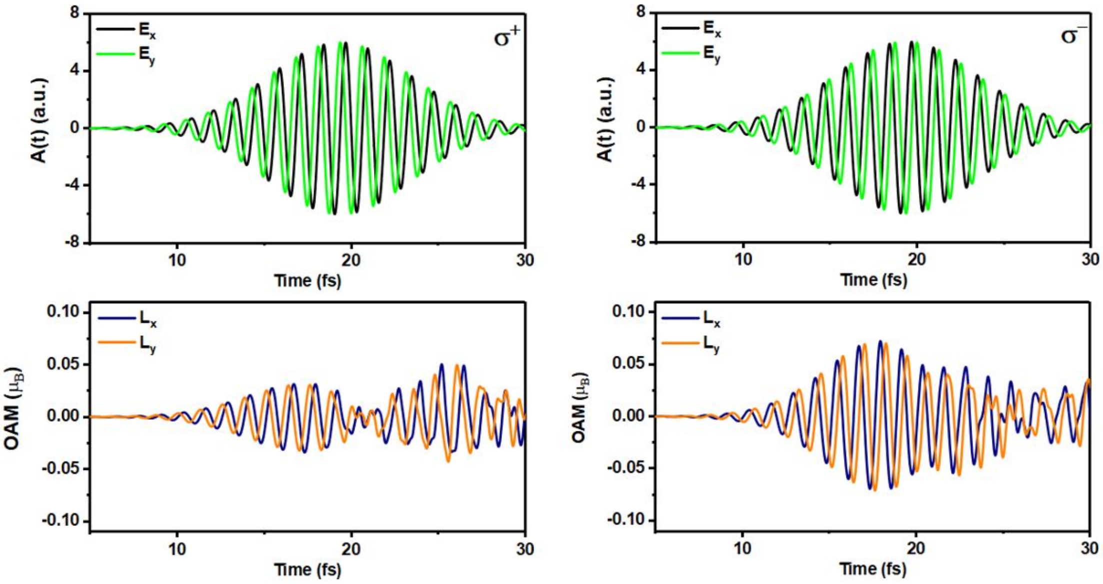

Figure S10 Panels on the left and right columns represent the cases of circularly σ^{+} and σ^{-} polarized lasers induced, respectively. From top to bottom, the panels show the time dependence of the amplitude vector potential in xy plane (A_x and A_y) of laser pulses and OAM of Cr atoms in CGT.

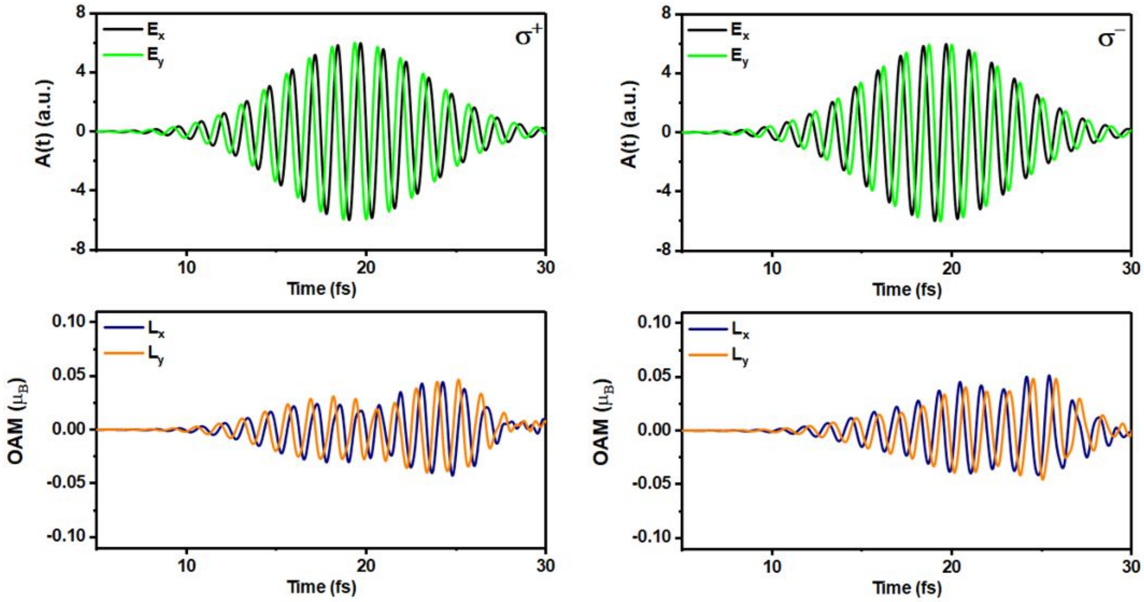

Figure S11 Panels on the left and right columns represent the cases of circularly σ^{+} and σ^{-} polarized lasers induced, respectively. From top to bottom, the panels show the time dependence of the amplitude vector potential in xy plane (A_x and A_y) of laser pulses and OAM of Cr atoms in CrI_3.

Table S1 The spin dynamics of FGT excited by circularly σ^+ and σ^- polarized lasers under the different parameters of laser pulses: Amplitude vector potential A(t), frequency (*f*), the full width at half maximum (FWHM), and fluence (F). The FWHM in first four rows of this table and the f in last four rows of this table are fixed.

A(t) (a.u.)	*f* (eV)	FWHM (fs)	F (mJ/cm^2)
70.58	0.54	9.68	28.29
28.74	1.36	9.68	28.29
12.00	3.27	9.68	28.29
9.60	4.08	9.68	28.29
6.00	3.27	9.68	7.06
5.50	3.27	14.51	8.90
5.00	3.27	19.35	9.80

References

1. Resta R. Magnetic circular dichroism versus orbital magnetization. *Phys. Rev. Res.* **2**, 023139 (2020).
2. Elliott P, Singh N, Krieger K, Gross EKU, Sharma S, Dewhurst JK. The microscopic origin of spin-orbit mediated spin-flips. *J. Magn. Magn. Mater.* **502**, 166473 (2020).
3. Dewhurst JK, Shallcross S, Elliott P, Eisebitt S, Schmising CvK, Sharma S. Angular momentum redistribution in laser-induced demagnetization. *Phys. Rev. B* **104**, 054438 (2021).
4. Dewhurst JKS, S. Elk code, elk.sourceforge.net *Elk code, elk.sourceforge.net*
5. Von Barth U, Hedin L. A local exchange-correlation potential for the spin polarized case. i. *J. Phys. C: Solid State Phys.* **5**, 1629 (1972).
